# Supplementary material for: Chromothripsis during telomere crisis is independent of NHEJ, and consistent with a replicative origin
Source: Genome Res. 2019 May;29(5):737–49. doi: 10.1101/gr.240705.118 (PMC6499312; doi:10.1101/gr.240705.118)
Supplement: Supplemental Material [file supp_gr.240705.118_Supplemental_file_1.zip › contigs/annotated_contigs/DB53/contig.15.DB53_length_2826_mean_cov_22.2505307856.docx]

**DB53_length_2826_mean_cov_22.2505307856**

```
CCTTGGCAGTCATTAATTCCTCCGTTA|TCCACAGCAAACCCATTTCCTTGCAACTGTTCTGTAATTTCCAGACCTCCTGAATTTTGGA
                            >chr8:77880404-77880870 - E=1e-234
GCCATATCACACAATCCCAATGCCAGGAACATAAGGACTTTTTAAAAGGTCTGTGCTTAGTCTCTTTAATAGGATTGAATATCACTTGC

GGCCTCATTGGTTTTATTTTTTTAAAATTAAGAAATCAGACTCCTTTTCTTTCCTATATATGTTGATTCCACACATGGGTGGATAACTG

GTAGCAAATTCAAAACCAACAGATTTCTTCGTATTTGAAACAATGACATTCATTCTTTTCCCTGGAATCAAAGCAGAAATATTAAGAGA

AACAAAAGTTTTCCCAAACCTAAATCCTTTTTCTTCAATCGTGCTTAAATTCACTCTTTTTTTACCCCAGAGTAAGGAAGTCAGGCAGA

GGGCTGGGGGAAATTCAGGAAGTGTCCATCTGTCAATTAATGGAGCTCC|TG|GGCTCACTGCAACCTCTGCCTACCGGCT|CTCAATG
                                                    >chr7:56606105-56606133 + E=2e-04
CCGGTAGCACTTTCCCATTCCCAGGTGTGCCAATTAAAAGGATCTCCAAATATTGTCAAAATTTCCCTGTGAGTCAAATTGCCCTCATT
 >chr17:68800276-68800426 + E=9e-78
TGAAAACCAAAAGCTTCAAATCCTCTAAAAATGCCCTGTTTTTGTAATAGTATG|CACCCATGCTGCAATGGAAGCTGGGTCAAGGAGC
                                                        >chr8:77874454-77874720 - E=2e-1
GGCATTGGAGCTGAAATTCAGGCTGCACTCTATGTGCCAAGTGTTGGACCCCAACACTGGGACTGTATCCACCCATAGAAGAAGGTGTC
47
TTTTTATAATTTTCATGAAGGTACTTCCATTTGGTCCACTCGTCATGCTTTTTATCTATGCGATTTAGTTTATCTCAAGAAGGAACCTT

CCGCTTATTGAGACAAAGTTGTCACCTGAGTTTAGGGGACCTGTGTTCTT|TACA|CGATCCTACTAATCCTACTAATGGGCATTTGCA
                                                   >chr8:79035228-79035307 - E=4e-35
CTTGCTGCTATTTCTCCCTTAAAAAACTAATGGCTCAAATAT|GCAC|GCATGTACATACAAATACCTGCATATTTAACA|AAACATAT
                                               >chr8:77987619-77987651 + E=7e-07 p=4e-
G|CATTAGACAAAACCAAACTCAAGTTATCAGTGGAAGAAATCTACCTGAGGAAAA|AC|TTCCTTCAGAAGAGAGAGAAGAGGGAAAA
02  >chr8:77880344-77880398 + E=4e-17                     >chr17:68799113-68799460 - E
TTTAAAGAGATAATAACCAATAATTATTTTAAAAGAATGAATGAAAATATAGACATGATGAAATTACATAAACAAAAGGCAAGATCCAA
=4e-196 p=0e+00
CTATATATTGTCTACAAAAAAAACATTAGAAATGTAAAACCTTAGAGTGACTAAAGTAAAAGGAACCACATCATAAACAAAAAACAAAA

AAGGTGGAATTACTATATTCATATCAGATAAATTAGACTGGATAAAAGGAATATTACCAGAAATAATGAGGGACATTACAAAATGATAA

CAAGGTTAGTTCACCAAAGATATAATTTAAATATGTATGCACCTTAAA|ACA|CACACACACACACTCACTTTAGAAAACGCATTGTAA
                                                 >chr17:68799723-68799914 - E=2e-102
AAGATAAAACAATCAGCAGAACCAGAATGAAAATTGACACAGACGGTTGAACAGCCAGAGAAATATTTTAAAATATGTATGATTAATAT

AATTATGCTGCTATATTATAAAAAGCTAGACACAAATGCATGAACAGATGGGAATTTC|AGCAG|CTAAAATTAATTCGGAAGAGTTAA
                                                           >chr8:81611511-81611585 - E=
CATTTACCACTTTTTCTACAAGATTTCTTCAAAATGGAAATGAAG|AACTATGGTGAATGAGTTCAAGAAAAAGGGATTTCAATTTTTT
4e-32 p=3e-02                                  >chr8:81031815-81032044 + E=3e-122 p=5e-0
ACTTTATACCTTGCTGTTCATGTAAAATTTGTTTCACACCATGTGAAAGAAAATATAAGCTTCTTTAAAATTAAAAGAATATATGGCCT
2
TTAAAACCACACAGTACTGGCATAAGAATAAACAAAACATTTGAAACAAGTCCAGAAATAGATCCACAAATATAAAGGAATTTAATGTA

T|GAAAATA|TGGGAATGCAGGCACATAATCTAGCCAA|CTC|ACTTTTCCATTATGCCTCTGTCGTCAGAATACTTCCGAGATTTGGA
  >chr8:77875356-77875391 - E=1e-08     >chr17:68549007-68549330 + E=1e-181
AGATAGTGACTCCACTTGGGATGGCTCTGAGGTCCTGCTCCTTCTTGGTATTTTGATAGAAAAGCCTTTTGCCACTCAAAGGGGAATTT

CTAAAAGAAACTTCAAACCACACAATTACTTTAAAAGGGTGATCACACAGTAATACATTGAATGATGCCTGGCCAGGATCTAACAAACT

TGTCTGTTTCCCTCTTTGTTGTGTCTGGGAGACAGAAAGACAGCCTGGCCATTCTCATGGCAAACTGTCATTAAATTATATCAAGTGTA

AATAGTTGTG|CAGATATCAACATAGGG|ACAATTGTCATATGCCCATTTGCCCAGCACTGTGTCTTTTATTAGTATACTCCACAAATG
                            >chr8:78542358-78542502 - E=4e-71
ATGATTAAATGCCTTCCATGTATTAGGCACCATGATGAATTTTAATCCTATGTCAGGTGCAGAAAGCGGTGTTAAATTTCTTGT|GC|A

CATACATAACACTTAGGATATGACACTAGGTATGTGTGGAAGGGTGGGCTGGAGGGTGGTTGCTAATAAAGATAATATGAGAGGCCACT
>chr8:85360113-85360474 - E=1e-201
CCTTCCCTTTTGGAGTCCCTTACCAGTAATTTAACATAAAACTGACATTATTCCCTGGTCACTCATGTATAAATTTAACTATATGTTAT

GACAAATGATCTAGTAAACAAAATCTTTCTCTCAAAGATTACCAGGCAGTGCTCTCTCTCTCTCTCCTCACTCTCGCTCTCTCTGTTGC

TAATACACAAAACCTTACATTTTCTTCCCCTAGCCCCTGTAATATTATACATTATAATGCGTTCACTTCCTCCATGTCAGTCTTTCTCA

CTAG
```
